# Predicting Visual Improvement After Macular Hole Surgery: A Combined Model Using Deep Learning and Clinical Features

**DOI:** 10.1167/tvst.11.4.6

**Published:** 2022-04-06

**Authors:** Alexandre Lachance, Mathieu Godbout, Fares Antaki, Mélanie Hébert, Serge Bourgault, Mathieu Caissie, Éric Tourville, Audrey Durand, Ali Dirani

**Affiliations:** 1 Faculté de Médecine, Université Laval, Québec, QC, Canada; 2 Département d'Ophtalmologie et d'oto-Rhino-Laryngologie – Chirurgie Cervico-Faciale, Centre Universitaire d'Ophtalmologie, Hôpital du Saint-Sacrement, CHU de Québec - Université Laval, Québec, QC, Canada; 3 Département d'informatique et de Génie Logiciel, Université Laval, Québec, QC, Canada; 4 Département d'ophtalmologie, Centre Hospitalier de l'Université de Montréal (CHUM), Montréal, Québec, QC, Canada; 5 Département de Génie Électrique et de Génie Informatique, Université Laval, Québec, QC, Canada

**Keywords:** artificial intelligence, visual acuity improvement, vitrectomy, macular hole

## Abstract

**Purpose:**

The purpose of this study was to assess the feasibility of deep learning (DL) methods to enhance the prediction of visual acuity (VA) improvement after macular hole (MH) surgery from a combined model using DL on high-definition optical coherence tomography (HD-OCT) B-scans and clinical features.

**Methods:**

We trained a DL convolutional neural network (CNN) using pre-operative HD-OCT B-scans of the macula and combined with a logistic regression model of pre-operative clinical features to predict VA increase ≥15 Early Treatment Diabetic Retinopathy Study (ETDRS) letters at 6 months post-vitrectomy in closed MHs. A total of 121 MHs with 242 HD-OCT B-scans and 484 clinical data points were used to train, validate, and test the model. Prediction of VA increase was evaluated using the area under the receiver operating characteristic curve (AUROC) and F1 scores. We also extracted the weight of each input feature in the hybrid model.

**Results:**

All performances are reported on the held-out test set, matching results obtained with cross-validation. Using a regression on clinical features, the AUROC was 80.6, with an F1 score of 79.7. For the CNN, relying solely on the HD-OCT B-scans, the AUROC was 72.8 ± 14.6, with an F1 score of 61.5 ± 23.7. For our hybrid regression model using clinical features and CNN prediction, the AUROC was 81.9 ± 5.2, with an F1 score of 80.4 ± 7.7. In the hybrid model, the baseline VA was the most important feature (weight = 59.1 ± 6.9%), while the weight of HD-OCT prediction was 9.6 ± 4.2%.

**Conclusions:**

Both the clinical data and HD-OCT models can predict postoperative VA improvement in patients undergoing vitrectomy for a MH with good discriminative performances. Combining them into a hybrid model did not significantly improve performance.

**Translational Relevance:**

OCT-based DL models can predict postoperative VA improvement following vitrectomy for MH but fusing those models with clinical data might not provide improved predictive performance.

## Introduction

Idiopathic full-thickness macular hole (MH) is a discontinuation of the neurosensory retina at the fovea and results in significant visual impairment including reduced visual acuity (VA) and metamorphopsia.^1^ Analysis of baseline optical coherence tomography (OCT) in patients with a MH can provide insight into surgical success and postoperative VA.^1^ OCT is commonly used in ophthalmology for multiple reasons: it allows noncontact, noninvasive, and easy-to-use cross-sectional images for clinical staging of various retinal diseases, including MH, by showing foveal and vitreous microstructures.

Pars plana vitrectomy with internal limiting membrane (ILM) peeling is commonly used to treat MH, with recent studies reporting the MH closure rate after a primary surgical procedure between 78% and 96%.^1^^–^^3^ Despite the high closure rate, functional outcomes after successful surgery are variable.^4^ Several authors tried to predict visual outcomes in closed MH based on clinical and imaging factors (e.g. MH duration, pre-operative VA, and MH size) or more specific OCT-defined parameters, such as macular hole index (MHI).^2^^,^^4^^,^^5^ Unfortunately, current predictive methods have some limitations that can cause inaccuracy and variability. For instance, pre-operative VA measurement is not always well standardized, MH duration based on symptoms onset is affected by recall bias and subjectivity, and OCT-based MH size and index ratios do not account perfectly for the hole asymmetry and overall hole shape. Moreover, measuring MH dimensions from OCT is time-consuming and requires expertise.

Therefore, other authors tried to predict visual outcomes in closed MH with automated three-dimensional (3D) analyses using pre-operative OCT B-scans to measure parameters, such as the base area, maximum base diameter, top area, maximum top diameter, minimum diameter, height, and the MHI.^6^^–^^8^ Despite its high predictive potential, using a 3D analysis instead of the traditional slice-based analysis suffers from some issues. Namely, modern scanners, such as the Cirrus high-definition (HD)-OCT (ZEISS, Dublin, CA), require 2 seconds to image a target cube. During this period, misalignment across slices can occur due to natural and involuntary movements of the subject's eyes.^9^ Although some eye motion correction in 3D-OCT B-scans exists, these inter-slice distortions and misalignments are a significant barrier to a full 3D analysis. Moreover, typically, macular cubes for 3D analysis (512 × 128 pixels) have lower resolution than traditional 2D OCT B-scans (750 × 500 pixels) using Cirrus 5000 HD-OCT, which gives less potential to increase neural network performances. In addition, clinicians routinely examine the OCT B-sans in a slice-by-slice manner. Thus, the ability to analyze and display information about MH in a slice-based manner is aligned with current clinical practices.

Promising deep learning (DL) models have been successfully applied to OCT B-scans to detect various ocular diseases, including diabetic retinopathy (DR), age-related macular degeneration (AMD), and MH.^10^^,^^11^ DL systems have also been used to predict antivascular endothelial growth factor treatment outcomes from pretreatment clinical images, showcasing their ability to also make predictions on the efficiency of a treatment from pretreatment knowledge.^12^ Because visual outcomes in MHs are affected by both morphological factors on OCT B-scans and other clinical factors, a hybrid model combining these is potentially helpful in predicting postoperative visual improvement.

This study aimed to assess the feasibility of DL methods to enhance the prediction of corrected visual acuity (CVA) increase at 6 months of ≥15 Early Treatment Diabetic Retinopathy Study (ETDRS) letters in closed MH after vitrectomy, by using pre-operative high-definition (HD)-OCT B-scans in addition to clinical data. To the best of our knowledge, this is the first time a combined model using DL and clinical features is used to try to predict visual outcomes in a closed MH.

## Methods

The chosen clinical ground truth was an improvement of ≥15 letters on ETDRS VA chart 6 months postoperatively. This threshold was previously considered to represent a clinically significant improvement in VA.^13^ We formulate this as a classification problem, where the DL model is asked to predict a binary outcome, that is whether CVA will improve by ≥15 letters 6 months after surgery. Predicting the precise number of letters gained postoperatively is subject to too much variation and instability, and has less interesting clinical implications (i.e. a CVA gain of 5 instead of 6 letters is not clinically significant). CVA was defined here as the best VA obtained using the patient's current refractive correction with or without pinhole.

This study was approved by the Institutional Review Board of the Centre Hospitalier Universitaire de Québec – Université Laval (2021-5371) and adheres to the tenets of the Declaration of Helsinki.

### Study Cohort

All consecutive patients operated for idiopathic full-thickness MH between 2014 and 2018 at the Centre Hospitalier Universitaire de Québec – Université Laval (Canada) were identified. All patients were operated by one of five vitreoretinal surgeons.

Hospital records were retrospectively reviewed to identify the patients with successful MH closure after primary vitrectomy. Only patients with an anatomic MH closure confirmed by HD-OCT B-scan following surgery were included. Data from patients with unclosed MHs was collected to assess our models’ ability to predict the visual outcome in those rare cases, but those were not used to train any of the classification models. Hole closure was defined as the absence of neurosensory retinal defect at the central fovea in all postoperative HD-OCT B-scans at 6 months.^5^ Exclusion criteria included patients with a follow-up of less than 6 months after the first surgery, history of a vitrectomy for any reason, and intra-operative use of a silicone oil tamponade or special techniques (e.g. free flap, inverted flap, retinal autografts, etc). Eyes with stage 1 MH, lamellar MH, MH secondary to other causes (e.g. trauma, AMD, type 2 macular telangiectasia, and retinal detachment), and eyes with ocular comorbidities that could potentially affect VA including high refractive or axial myopia (≥6 diopters of myopia or axial length ≥26 mm) were excluded. In patients with bilateral MH on initial presentation, only the first operated eye was included. Eyes with significant cataract prior to surgery and eyes that developed clinically significant cataract during the 6 months follow-up were excluded.

### Dataset Preparation and Feature Collection

We reviewed the records of all eyes operated for MH with a vitrectomy, ILM peeling, and gas or air tamponade. The type of tamponade used was at the discretion of the surgeon with C_3_F_8_ often used in more complex cases. All patients were advised to position face-down after surgery for 5 to 7 days.

Pre-operative data included age, sex, lens status, myopia, MH duration defined as the duration between the first reference and the time of surgery,^14^ baseline CVA, and MH size. MH size was measured as the minimum hole width or the narrowest aperture size in the middle retina, as defined by the Vitreomacular Traction Study Group^15^ on initial presentation. MH measurements were performed by the same person (author A.L.) and validated by a retina specialist (author A.D.) with consensus when discordance.

To reduce overfitting and to gain a better understanding of the clinical importance of each input feature, we decided to select the following features as the main clinical factors predicting final CVA according to the previous literature: baseline CVA, MH size, MH duration, and pseudophakic status.^2^^,^^4^^,^^5^ Operative data included surgical technique, type of dye, and type of tamponade used. Postoperative data included CVA at 6 months postoperatively. The CVA originally reported on the Snellen scale was converted to logarithm of the minimal angle of resolution (logMAR).^16^ Lens status and HD-OCT B-scans were recorded at baseline and at 6 months postoperatively.

All patients had undergone HD-OCT imaging using Cirrus 5000 HD-OCT (Carl Zeiss Meditec, Jena, Germany) with 30 degrees centered on the fovea. The individual HD-OCT B-scans horizontal and vertical lines were 750 × 500 pixels. Each patient's image data set was exported as a folder of anonymized non-compressed TIFF files. Our data set did not include images with improper positioning, low signals, or strong motion artifacts causing misalignment and blurring; no images were excluded.

We make our data set and codebase publicly available to stimulate and ease further research on the topic.

### Training and Validation of DL-Based Model

We randomly split the 121 patients of the data set into training, validation, and test subsets, each respectively containing 83 (69%), 21 (17%), and 17 (14%) patients. As their names indicate, the training set was used to train DL models, the validation to select the best performing model, and the test set to evaluate the performance of the chosen model in comparison with baselines.

We selected the CBR-Tiny^17^ DL model for our experiments because it displays competitive performances on medical imaging data sets despite its simplicity. During training, we leveraged the fact that we have two HD-OCT B-scans (i.e. horizontal and vertical lines) for each patient to artificially double our training samples. At inference time, we took the average of the prediction provided by both HD-OCT B-scans associated with each patient. We applied randomized data augmentation, randomly rotating, flipping horizontally, and adjusting the brightness and contrast of every image in a training batch. All augmented images were then resized to the 224 × 224 pixels range and then normalized using the mean and standard deviation values from the ImageNet data set.^18^ Every vision model was trained using the binary cross-entropy loss with a batch size of 32, using the Adam^19^ optimizer with a learning rate of 0.0001 for a maximum of 1000 gradient steps. We tested the model's performance against the validation set every 50 steps and retained the model with the highest validation area under the receiver operating characteristic curve (AUROC).

Visualization heatmaps were generated by Gradient-weighted Class Activation Mapping (Grad-CAM). This was done to help understand the areas of interest on the HD-OCT B-scans which were considered by the DL model.

### Regression Model

To provide a baseline for comparison of the DL model's performance, we trained a logistic regression model on clinical features. Hyperparameters used for the logistic regression, such as the regularization coefficient, were selected using 10-fold cross-validation over the training set. Because our logistic model was trained using cross validation, it did not require a held-out validation set like the DL model. Accordingly, we trained the regression model on samples from both the DL training and validation sets, using the held-out test set to assess performance. Specifically, this meant that our logistic regression model was trained on clinical data of the 104 patients of the testing and validation subsets and tested on the same 17 patients as the DL model. As stated above, the retained features for each patient were: baseline CVA, MH size, MH duration, and pseudophakic status. Before being fed to the model, each patient's clinical data was standardized using the mean and standard deviation of each feature computed on the training set.

Our implementation of the regression model used the *LogisticRegressionCV* model from scikit-learn^20^ with Python version 3.8.

### Hybrid Model

**Figure 1.**
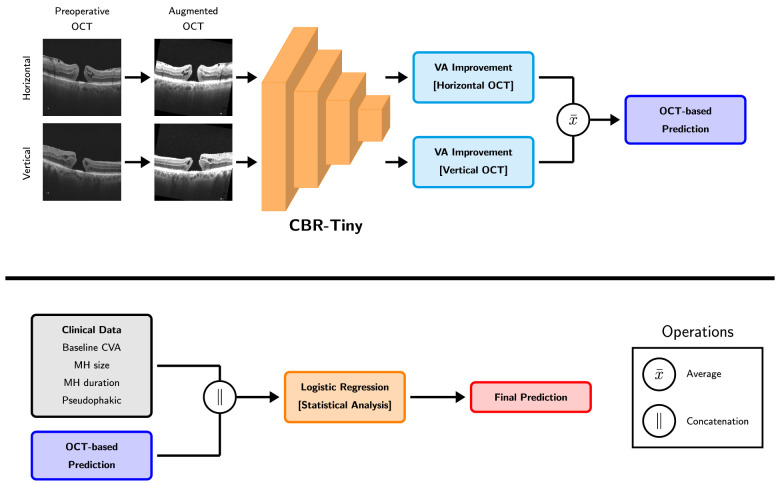
Overview of our proposed hybrid model. (*Top*) Illustration of the extraction of the OCT-based prediction from the trained DL model. (*Bottom*) Flow-chart representing the combination of clinical data and OCT-based data to predict the clinical ground truth.

From the fully trained deep vision model, we extracted the model's logistic prediction for a patient's HD-OCT B-scans and concatenated it to the patient's clinical data. This clinical data augmented with the vision model's predictions were then used to train another logistic regression, yielding an easy-to-implement way to combine HD-OCT B-scans and clinical data. Hyperparameters for this hybrid model were selected according to the same procedure as our regression model from clinical data only. An overview of this proposed hybrid model can be seen in Figure 1.

Many techniques exist to handle multimodal data like our images and clinical features. These techniques, usually classified under late, medium, or early fusion,^21^ represent an increasing usage of DL, with early fusion meaning the whole multimodal data is fed to a neural network and late fusion meaning only the DL model's prediction is kept. We chose late fusion as this preserves the interpretability. Indeed, because the final prediction is provided by logistic regression, one can compute each feature's importance according to the model, including for the DL model prediction. Albeit potentially less performant, late fusion still represents an interesting avenue in healthcare tasks, where interpretation of the model is highly valuable.

### Statistical Analysis

After training and testing our hybrid model, we obtained the predictive output of combining each HD-OCT B-scan with the corresponding patient's clinical data. The confusion matrices were used to calculate the overall accuracy of prediction of visual outcome (CVA gain ≥15 letters) at 6 months. AUROC was used to evaluate the reliability of the model in predicting CVA gain. Other outcome measures included F1 scores, accuracy (ACC), sensitivity (SN), specificity (SP), positive predictive value (PPV), and negative predictive value (NPV).

For both regression-based models (i.e. clinical data only and hybrid), we extracted feature importance for each input feature by computing the ratio between the feature's weight and the sum of all model feature weights.

Data for patient baseline characteristics are presented as mean ± standard deviation for continuous variables and as frequencies (percentages) for categorical variables. Statistical analyses were performed using Python (version 3.8.10; Python Software Foundation) with the Numpy library.^22^ Statistical significance was set at α = 0.05.

### Cross-Validation

Due to the small size of our held-out test set, we performed five-fold cross-validation with all three of our proposed models. For the regression model based solely on clinical features, we split the training set into five mutually exclusive groups, using each group as test set and the four others as training set for a total of five different runs. We repeated this five-fold cross-validation with five different group separations for a total of 25 runs. For the DL model, which also required a validation set for model selection, we repeated the process by using each of the four training folds as validation set, for a total of 100 runs of the DL model. For each DL model run, a corresponding hybrid model run was done by using the trained DL model and its corresponding training and testing sets for regression.

### Performance Assessment on Unclosed MH

Although all our models were trained using our data set that contains closed cases of MH, it is known that some MH cases may remain unclosed after surgery. To assess our model's ability to predict the visual outcome of such unclosed cases, we tested the performance of our trained hybrid models on the 16 unclosed MH cases of our cohort.

## Results

Characteristics of eyes included in the different subsets (i.e. training, validation, and held-out test sets) are shown in Table 1. Among the 121 patients included (242 HD-OCT B-scans), 88 (73%) were women and the mean age was 67 ± 8 years.

**Table 1.** Characteristics for Patients of the Different Splits in the Data Set

	Training Set (*n* = 104)	Test Set (*n* = 17)	Total Set (*n* = 121)	*P* Value
**Age**, years ± SD	66 ± 8	69 ± 7	67 ± 8	0.12
**Sex**				0.84
Female, *n* (%)	76 (73)	12 (71)	88 (73)	
Male, *n* (%)	28 (27)	5 (29)	33 (27)	
**Baseline CVA**, letters ± SD	50 ± 15	51 ± 18	50 ± 16	0.83
**MH size**, µm ± SD	357 ± 171	256 ± 101	343 ± 167	0.002
**MH duration**, weeks ± SD	11 ± 10	10 ± 5	11 ± 9	0.53
**Pseudophakic**, *n* (%)	18 (17)	4 (24)	22 (18)	0.54
**Phacovitrectomy**, *n* (%)	1 (1)	0 (0)	1 (1)	0.68
**CVA at 6 months**, letters ± SD	66 ± 12	66 ± 11	66 ± 12	1.00
**CVA gain ≥15 letters**, *n* (%)	52 (50)	8 (47)	60 (50)	0.83
**Stage of MH**				0.81
Stage 2, *n* (%)	16 (15)	3 (18)	19 (16)	
Stage 3, *n* (%)	64 (62)	10 (59)	74 (61)	
Stage 4, *n* (%)	24 (23)	4 (24)	28 (23)	

MH, macular hole; CVA, corrected visual acuity; SD, standard deviation.

Table 2 presents the comparison between the two groups (CVA gain ≥15 letters versus <15 letters) in terms of demographic and clinical features. There was no difference in the type of tamponade and dye used between the two groups (*P* = 0.98 and *P* = 0.11, respectively), which ensures comparability of confounding factors. Vitrectomy with sulfur hexafluoride (SF_6_) gas tamponade and indocyanine green (ICG) was most commonly performed. Baseline CVA and MH size were statistically different between the two groups, but MH duration and pseudophakic status were not.

**Table 2.** Comparison Between the Two Groups (CVA Gain ≥15 Letters Versus <15 Letters) in Terms of Demographic and Clinical Features

Features	CVA Gain ≥15 Letters (*n* = 60)	CVA Gain <15 Letters (*n* = 61)	Total Set (*n* = 121)	*P* Value
**Age**, years ± SD	67 ± 15	67 ± 14	67 ± 15	1.00
**Sex**				0.17
** **Female, *n* (%)	47 (78)	41 (67)	88 (73)	
** **Male, *n* (%)	13 (22)	20 (33)	33 (27)	
**Baseline CVA**, letters ± SD	42 ± 15	59 ± 10	50 ± 16	<0.0001
**MH size**, µm ± SD	394 ± 183	293 ± 131	343 ± 167	0.0008
**MH duration**, weeks ± SD	11 ± 12	10 ± 7	10 ± 10	0.58
**Pseudophakic**, *n* (%)	15 (25)	7 (12)	22 (18)	0.05
**Phacovitrectomy**, *n* (%)	0 (0)	1 (2)	1 (2)	0.32
**CVA at 6 months**, letters ± SD	70 ± 9	62 ± 12	66 ± 12	<0.0001
**Stage of MH**				0.53
** **Stage 2, *n* (%)	10 (17)	9 (15)	19 (16)	
** **Stage 3, *n* (%)	35 (58)	39 (64)	74 (61)	
** **Stage 4, *n* (%)	15 (25)	13 (21)	28 (23)	
**Surgery procedure**				
** **Vitrectomy with ILM peeling, *n* (%)	60 (100)	61 (100)	121 (100)	0.99
**Tamponade used**				0.98
** **SF_6_, *n* (%)	53 (88)	54 (89)	107 (88)	
** **C_3_F_8_, *n* (%)	7 (12)	7 (12)	14 (12)	
**Dye used**				0.11
** **ICG, *n* (%)	43 (72)	51 (84)	94 (78)	
** **TB, *n* (%)	17 (28)	10 (16)	27 (22)	

MH, macular hole; ILM, internal limiting membrane; CVA, corrected visual acuity; ICG, indocyanine green; TB, trypan blue; SD, standard deviation.

Using only logistic regression on clinical features, in the training set, the AUROC was 80.6 with an F1 score of 78.2. On the held-out test set, the AUROC was 80.6 with an F1 score of 79.7. For the DL model, in the training set, relying solely on the HD-OCT B-scans, the AUROC was 77.3 ± 10.3 with an F1 score of 67.1 ± 28.9. On the held-out test set, the AUROC was 72.8 ± 14.6 with an F1 score of 61.5 ± 23.7.

For our hybrid model, in the training set, the AUROC was 84.09 ± 1.58 with an F1 score 78.0 ± 1.7. On the held-out test set, the AUROC was 81.9 ± 5.2 with an F1 score of 80.4 ± 7.7. Performances of the models on the held-out test and using cross-validation are shown in the Table 3. All reported results consist of 95% confidence intervals (CI) based on 10 independent training runs. Our models do not require a validation set because the best parameters are selected according to 10-fold validation, hence the absence of validation results.

**Table 3.** Performances of the Models on the Held-Out Test and Using Cross-Validation

Models	F1 Scores	AUROC	ACC	SP	SN	PPV	NPV
**Clinical**							
Train	**78.2**	79.3	76.0	**88.5**	**71.7**	**80.0**	72.9
Test	79.7	80.6	**80.2**	89.7	72.3	**80.0**	**81.8**
**OCT-based DL**							
Train	67.1 ± 28.9	77.3 ± 10.3	69.8 ± 3.6	76.9 ± 25.2	65.4 ± 15.6	57.6 ± 14.4	**81.4** ± 9.0
Test	61.5 ± 23.7	72.8 ± 14.6	63.9 ± 13.2	70.8 ± 30.2	60.2 ± 17.9	60.2 ± 15.4	76.9 ± 15.4
**Hybrid**							
Train	78.0 ± 1.7	**84.1** ± 1.6	**76.9** ± 4.2	79.0 ± 16.8	**76.4** ± 26.8	74.2 ± 9.4	78.8 ± 7.6
Test	**80.4** ± 7.7	**81.9** ± 5.2	78.7 ± 2.9	**91.3** ± 15.9	67.8 ± 26.9	77.4 ± 4.3	80.8 ± 6.7
**Clinical cross-validation**							
Train	**77.0** ± 2.1	**82.4** ± 2.7	**76.5** ± 5.3	84.7 ± 9.9	64.2 ± 19.9	72.6 ± 9.5	**83.2** ± 6.9
Test	**81.0** ± 7.1	**81.5** ± 11.2	**80.3** ± 10.8	**97.2** ± 5.0	55.4 ± 23.2	70.4 ± 11.0	86.7 ± 5.9
**OCT-based DL cross-validation**							
Train	74.0 ± 3.7	75.3 ± 6.9	73.5 ± 7.3	**85.2** ± 8.5	53.8 ± 21.5	66.8 ± 9.0	79.5 ± 7.3
Test	76.3 ± 6.8	74.8 ± 11.1	74.9 ± 10.3	87.3 ± 11.2	57.2 ± 25.8	70.3 ± 13.5	81.3 ± 14.6
**Hybrid cross-validation**							
Train	76.8 ± 2.6	82.2 ± 3.2	76.4 ± 5.6	80.6 ± 9.8	**70.1** ± 20.0	**75.7** ± 10.5	80.0 ± 6.0
Test	80.1 ± 7.6	81.7 ± 10.6	79.3 ± 10.7	92.4 ± 9.3	**60.2** ± 23.6	**72.3** ± 12.6	**90.6** ± 10.3

DL, deep learning; AUROC, area under the receiver operating characteristic curve; ACC, accuracy; SP, specificity; SN, sensitivity; PPV, positive predictive value; NPV, negative predictive value.

Best means are highlighted.

**Figure 2.**
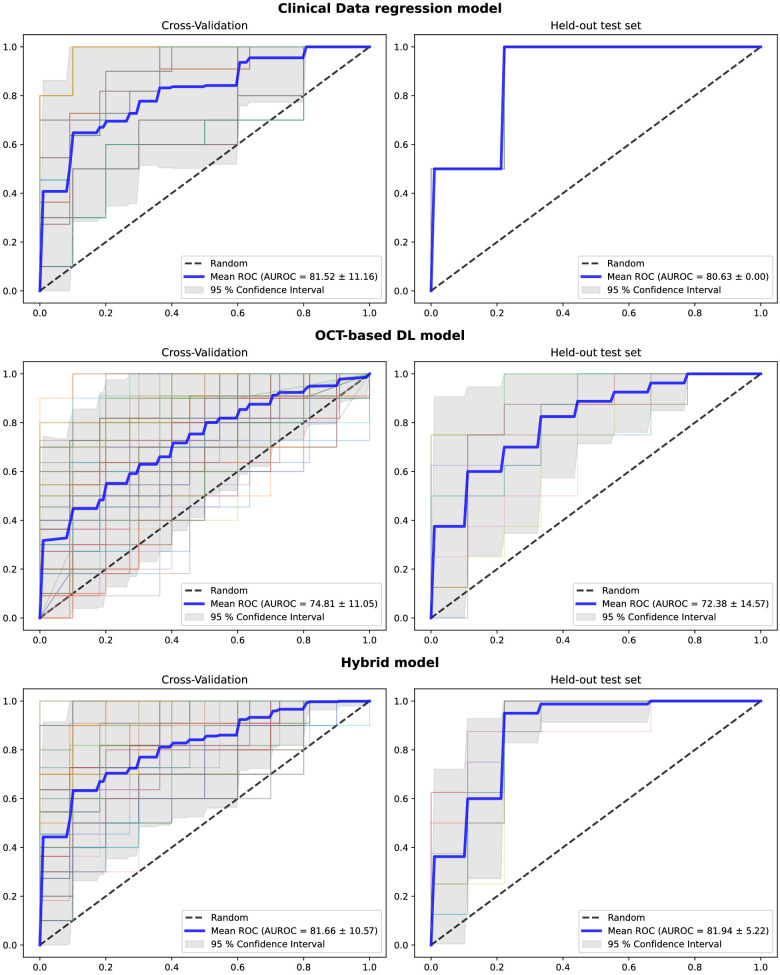
The receiver operating characteristic (ROC) curves on the cross-validation and held-out test set for all three models. We report the mean value and a 95% CI computed from 100 independent runs for the DL and hybrid models and 25 runs for the clinical data regression model. For each independent run, we plotted the corresponding ROC curve in a different color.

The receiver operating characteristic (ROC) curves of all independent runs are presented for the clinical, OCT-based DL, and hybrid model in Figure 2. These results indicate that our hybrid model can provide interesting prediction of postoperative visual improvement with discriminative performances, but this is not significantly better than the clinical data-only logistic regression model.

**Figure 3.**
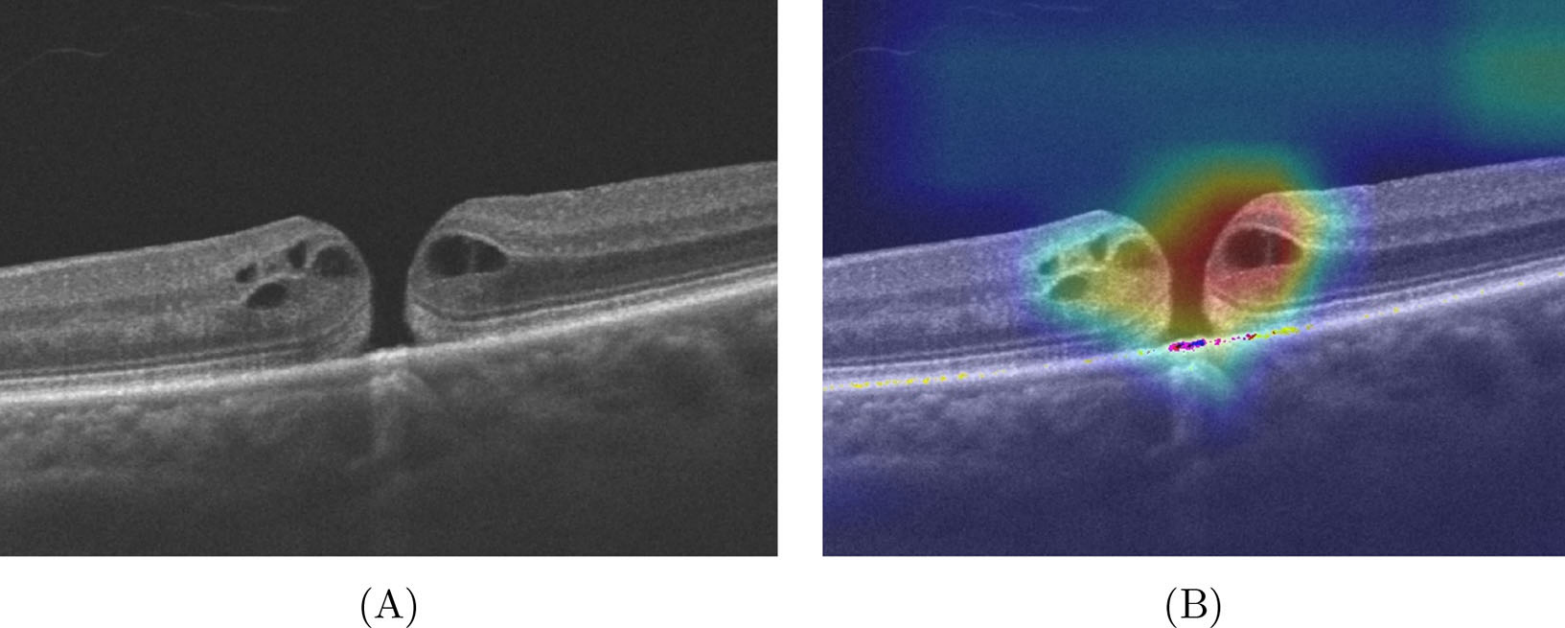
Visualization heatmap for prediction of the clinical ground truth. The heatmap was generated by Gradient-weighted Class Activation Mapping (Grad-CAM). The heatmap highlights the pathological area (fovea) as being most important for accurate prediction of CVA improvement after surgery in HD-OCT B-scans. (**A**) Original image. (**B**) Grad-CAM.

Heatmaps illustrated the most important region for decision making in our OCT-based DL model (Fig. 3). The fovea was identified as the most critical region for prediction of the clinical ground truth, suggesting that general MH morphology is considered by the model.

**Figure 4.**
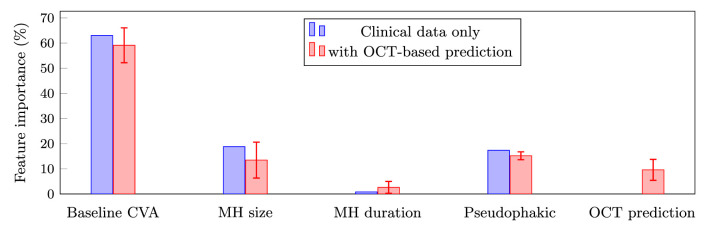
Feature importance ratio (%) attributed by the regression models with and without the addition of the OCT-based prediction. For each feature, we reported the average and standard deviation computed from 10 independent runs. The standard deviation is 0 for all features when using only clinical data because the clinical features are always strictly the same in this case. MH, macular hole; CVA, corrected visual acuity.

For both regression-based models (i.e. clinical data only and hybrid), we reported feature importance for both models in Figure 4. We see that, for both models, the baseline CVA was the most important feature, being assigned 63.0 ± 0.0% of the model's weight for clinical data only and 59.1 ± 6.9% in the hybrid model. Notably, the OCT prediction only contributes moderately to the hybrid model's output, as its importance ratio is 9.6 ± 4.2%.

**Figure 5.**
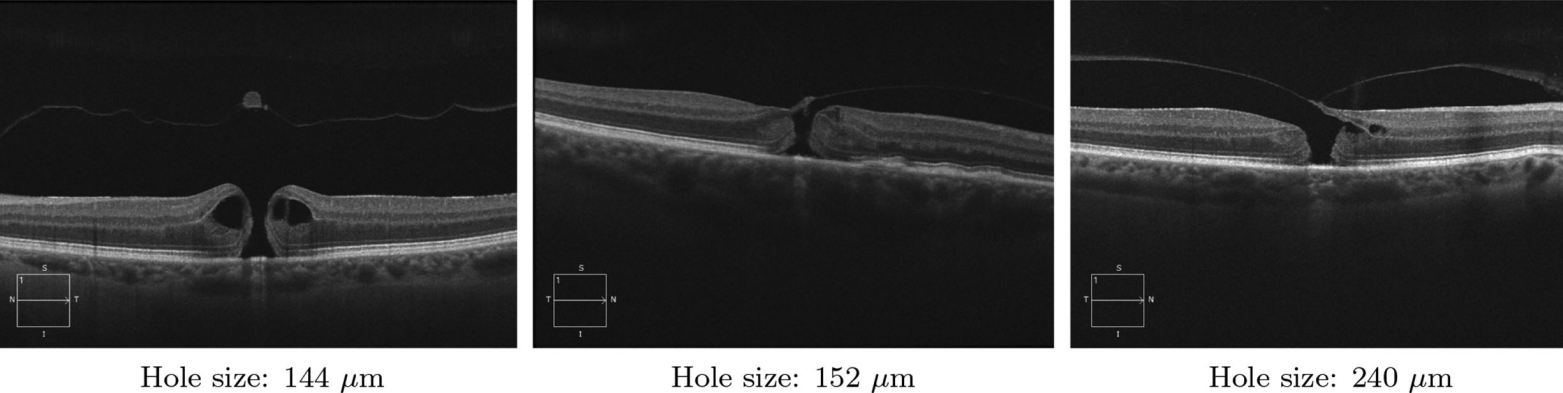
Horizontal HD-OCT of the three most difficult cases to predict on the held-out test with the hybrid model.

We also reported in Table 4 the differences in the pre-operative and postoperative factors between eyes predicted correctly and incorrectly by the hybrid model. It was more difficult for the hybrid model to predict the accurate visual outcome when the MH size was smaller (180 ± 38 versus 280 ± 103 µm; *P* = 0.01). MH size was higher in the training set compared to the test set (357 ± 171 and 256 ± 101 µm, respectively; *P* = 0.002) which may explain the lower performances due to less examples of small MH in the training of the DL model. The three most difficult cases to predict are shown in Figure 5.

**Table 4.** The Differences in the Pre-Operative and Postoperative Factors Between Eyes Predicted Correctly and Incorrectly by the Hybrid Model

	Eyes Predicted Correctly (*n* = 13)	Eyes Predicted Incorrectly (*n* = 4)	*P* Value
**Age**, years ± SD	70 ± 7	66 ± 3	0.13
**Sex**			0.83
Female, *n* (%)	9 (69)	3 (75)	
Male, *n* (%)	4 (31)	1 (25)	
**Baseline CVA**, letters ± SD	51 ± 19	49 ± 11	0.80
**MH size**, µm ± SD	280 ± 103	180 ± 38	0.01
**MH duration**, weeks ± SD	9 ± 7	9 ± 4	1.00
**Pseudophakic**, *n* (%)	2 (15)	2 (50)	0.15
**Phacovitrectomy**, *n* (%)	0 (0)	0 (0)	1.00
**CVA at 6 months**, letters ± SD	67 ± 7	66 ± 3	0.69
**CVA gain ≥15 letters**, *n* (%)	6 (46)	2 (50)	0.90
**Stage of MH**			0.05
Stage 2, *n* (%)	1 (8)	2 (50)	
Stage 3, *n* (%)	9 (69)	1 (25)	
Stage 4, *n* (%)	3 (23)	1 (25)	

MH, macular hole; CVA, corrected visual acuity; SD, standard deviation.

MH size is smaller in the eyes predicted incorrectly.

Table 5 shows the performances of our hybrid model when applied to unclosed MH cases in comparison to our held-out test set of closed MH cases. Overall, we noted no significant performance difference when testing on unclosed MH cases. F1 scores, AUROC, and ACC for the hybrid model tested with closed MH versus unclosed MH: 80.4 ± 7.7, 81.9 ± 5.3, and 78.7 ± 2.9 versus 77.8 ± 1.4, 79.3 ± 3.1, and 79.4 ± 1.9.

**Table 5.** Performances of the Hybrid Model on the Held-Out Test on Unclosed Versus Closed Macular Holes After the First Vitrectomy

Hybrid Model	F1 Scores	AUROC	ACC	SP	SN	PPV	NPV
**Test set (closed MH)**	80.4 ± 7.7	81.9 ± 5.2	78.7 ± 2.9	91.3 ± 15.9	67.8 ± 26.9	77.4 ± 4.3	80.8 ± 6.7
**Unclosed MH set**	77.8 ± 1.4	79.3 ± 3.1	79.4 ± 1.9	100.0 ± 0.0	72.7 ± 2.0	69.7 ± 1.0	100.0 ± 0.0

DL, deep learning; AUROC, area under the receiver operating characteristic curve; ACC, accuracy; SP, specificity; SN, sensibility; PPV, positive predictive value; NPV, negative predictive value.

The performances are similar which indicates that our hybrid model well generalized to MH that failed to close after the first vitrectomy.

## Discussion

In this study, we developed a hybrid model that used DL on pre-operative HD-OCT B-scans and logistic regression on clinical features to predict CVA improvement ≥15 ETDRS letters at 6 months in closed MH after a vitrectomy surgery. Despite the limited total number of OCT scans (*n* = 242), both our models based on clinical data and OCT scans had good discriminative performances. Combining both models into a hybrid model using late fusion yielded marginally better performance, but the improvement was not statistically significant when considering 95% CIs (clinical data only: AUROC of 80.6 and F1 score of 79.7 versus hybrid model: AUROC of 81.9 ± 5.2 and F1 score of 80.4 ± 7.7); conclusions were similar when we used cross-validation. Given the fact that the hybrid model does not attribute much weight to the prediction provided by the HD-OCT scans (i.e. 9.6 ± 4.2%), it appears that clinical data regression and OCT-based DL are correlated. Much of the information provided by the OCT-based DL model is likely already represented in the clinical data only model as MH morphology in baseline CVA, size, and duration of the MH. Moreover, we tested our hybrid model on the unclosed MHs of our cohort and the results were similar compared to the held-out test with the closed MHs (Table 5), which highlights that the visual prediction of our model is able to generalize to MH that failed to close after the surgery. Thus, the closure of the MH may not be a crucial data to obtain when we have pre-operative OCT and clinical variables (especially VA). This may be explained by the fact that information extracted from OCT provides information on the closure of the MH (e.g. large MH size makes closure of the hole less likely). In this way, training a model with a MH that has not closed may not be essential.

Our model based on OCT-based DL is a new and promising alternative to clinical data for prediction of outcomes after surgery. This can lead to new applications in ophthalmology in the future. However, our results need to be contextualized. The difficulty of the task is greater than the DL image classification that detects retinal diseases on OCT as MH, where the AUROC is often much higher (e.g. 97.8%).^10^ Our task is difficult even for an experienced retinal specialist, it is therefore expected that our combined prediction model cannot reach near-perfect accuracy. Other studies used DL technology to predict anatomic outcome after MH surgery.^23^^,^^24^ Despite similarities between these two tasks, the task to predict MH status (closed or open) after the vitrectomy seems to be easier. MH closure essentially amounts understanding the shape of the MH, whereas predicting visual outcome also requires understanding how one MH's shape and its restoration impacts the patient's VA. The postoperative integrity of some layers of the retina like the external limiting membrane (ELM) and the ellipsoid zone are correlated with postoperative VA.^25^ The DL model must then include the prediction of arrangement and reorganization of these layers to predict the VA gain.

These results are interesting in real-world clinical settings. Prediction of postoperative visual outcomes can allow clinicians to give a more accurate prognosis to patients and help alleviate their anxiety. This could eventually help ophthalmologists make better surgical decisions in patients with a poor visual prognosis. Our model is built on standard surgical methods for primary MHs with no other pathologic disease that could potentially affect VA, but future investigations could help to predict vision in eyes with concomitant other eye diseases, such as diabetic macular edema. Although the difficulty of this task is greater, advanced work in DL in connection with this pathology could be favorable.^11^

OCT imaging is a useful way of measuring various aspects of MH morphology with high precision and reproducibility.^26^ Some OCT-defined parameters have been used as prognostic factors of visual outcomes in closed MH after surgery.^6^^–^^8^ However, these OCT-defined parameters were evaluated individually in previous studies. The accuracy and applicability of these prediction algorithms using single factors are limited, whereas visual outcomes after MH surgery are influenced by multiple factors. Clinical data are also crucial to better understand VA improvement. Three characteristics are important for prediction and included in our model: pre-operative VA, MH duration, and MH size.^2^^,^^4^^,^^5^ Eyes with better baseline VA generally obtain better postoperative VA, whereas eyes starting with worse VA have more potential VA gain.^27^^–^^29^ Moreover, MHs of shorter durations are associated with better visual outcomes as the integrity of the macular structure and ELM are better preserved.^25^ Furthermore, an inverse correlation between MH size and postoperative VA is well recognized, with larger MH typically associated with worse visual outcomes.^30^^,^^31^ Lens status was also included (i.e. phakic or pseudophakic) to limit the effect of progressive cataract development in a patient with a phakic lens on postoperative VA. In our study, we propose a straightforward way to integrate DL on OCT images and relevant clinical data to make accurate predictions of postoperative visual improvement.

Concurrently to our work, Obata et al. also investigated the task of predicting VA after MH surgery, finding that a DL model on OCT scans performed better than logistic regression from clinical data.^32^ Although closely related to our approach, their work differed in the prediction task given to the model. Indeed, whereas our approach aimed to provide binary classification (whether the VA will increase by 15 ETDRS at 6 months or not), their task was a multiclass classification (whether a patient's VA will be within four different ranges after surgery). The latter was an intuitively harder task and was probably the reason behind their lower performances (precision of 46% for the DL model) compared with ours. Moreover, they compared their DL model to a multiple linear regression model in which clinical data included pre-operative VA, MH size, and age with no inclusion of pseudophakic status. In our study, 15.2 ± 1.6% of the model's weight of the hybrid model was related to pseudophakic status. This is likely less relevant in Obata et al.'s work given that they performed phacovitrectomy in 99% (236/238) of patients with phakic lenses.

In future works, our approach can probably benefit from using transfer learning. In transfer learning, a DL model is initially trained on an auxiliary task usually related to the task at hand. Using the pretrained model's weights as a starting point, the fully trained model's performance can then be improved with a smaller sample size for the target task. This is of particular interest in the medical field where imaging data is more difficult to obtain in large quantities.^33^^,^^34^ Another interesting research direction would be to consider other fusion schemes than late fusion^21^ for the hybrid model. Indeed, whereas late fusion enjoys the desirable property of preserving interpretability, it still represents a very limited source of information sharing between the clinical data and the OCT models. Other approaches fall into the early and medium fusion categories,^21^ potentially increasing the hybrid model's performance at the cost of losing interpretability. Recently, one such early fusion model for prediction of the anatomical outcome of MH surgery was proposed by Xiao et al.^35^ who found that a hybrid model had better performance than a model based exclusively on OCT scans and clinical data (AUROC of 90.4, 80.4, and 79.7, respectively). We hypothesize that significant gain in performance found when compared to our work is attributable to the easier nature of the anatomic task as well as their hybrid model's increased capacity. To this end, we hope that making our data set and codebase publicly available will enable other researchers to further progress on the proposed task, by testing the above proposed methods or through their own alternatives.

This study has limitations. As seen in Table 1, the DL model trained on MH with larger diameters than in the test set (*P* = 0.02) and ended up making more mistakes on smaller MH sizes, as seen in Table 4. This discrepancy in the MH sizes between sets was an artifact of our random sampling procedure to separate the train and test patients. In our procedure, we randomly assigned the MHs in the groups, ensuring the visual outcomes in both sets were as similar as possible while attempting to also do the same for clinical features. Considering the high variance in patients across all features and the small size of the test set (17 patients), one can hardly expect to have feature distributions that are as similar as the two sets we used. Moreover, it is well known that baseline CVA is the most important clinical predictor (hybrid model weight of baseline CVA: 59.1 ± 6.9%), and this information was separated adequately between train and test sets.^4^ We considered our random split to be as accurate as possible given our limited number of patients. It is to be expected that the model will be more accurate on patients that are similar to what it was trained on, so the lower occurrence of large MH sizes in the training set probably explains why the model's performance worsens on smaller MH sizes. One way to possibly alleviate this performance discrepancy would be to use stratification based on the MH size attribute. We chose not to use stratification because it would have significantly reduced the amount of data available to train on, which was already a bottleneck for our DL model.

Moreover, cataract surgery can have a significant impact on the results, but we took all the measures to limit its effect. Eyes with significant cataract prior to surgery and eyes that developed clinically significant cataract during the 6 months of follow-up were excluded. Moreover, we selected the prediction at 6 months to minimize the development of cataract in phakic eyes. In addition, few eyes had phacovitrectomy (1 eye) and no patients had a cataract surgery during the follow-up.

Our model had a relatively small sample size, issued from a single center. Perspectives for external validation are underway. Currently, our hospital center does not have new data to assess external validity and no public data set contains OCT with the necessary clinical information as CVA.^36^ That said, this is a preliminary study to evaluate the feasibility of predicting postoperative CVA improvement with DL methods. Further studies are also needed to determine the mechanisms guiding model prediction.

In conclusion, despite the limited total number of OCT scans, both models from clinical data and OCT scans had good discriminative performances. Combining both models into a hybrid model yielded marginally better performance, although the improvement was not statistically significant. Our models allow prediction of visual improvement after successful MH closure surgery. These models could eventually help ophthalmologists for surgical planning of MH surgery and better care for patients with tailored visual prognoses.

## Appendix

Amended April 15, 2022: A few very minor style changes were made to the text.
